# Microbial Existence in Controlled Habitats and Their Resistance to Space Conditions

**DOI:** 10.1264/jsme2.ME14032

**Published:** 2014-08-12

**Authors:** Kasthuri Venkateswaran, Myron T. La Duc, Gerda Horneck

**Affiliations:** 1 Biotechnology and Planetary Protection Group, California Institute of Technology, Jet Propulsion Laboratory, Pasadena, CA 91109, USA; 2 Institute of Aerospace Medicine, DLR (German Aerospace Center), D 51170, Köln, Germany

**Keywords:** Closed habitat, International Space Station, PLANET PROTECT, BOSS, Microbial Observatory

## Abstract

The National Research Council (NRC) has recently recognized the International Space Station (ISS) as uniquely suitable for furthering the study of microbial species in closed habitats. Answering the NRC’s call for the study, in particular, of uncommon microbial species in the ISS, and/or of those that have significantly increased or decreased in number, space microbiologists have begun capitalizing on the maturity, speed, and cost-effectiveness of molecular/genomic microbiological technologies to elucidate changes in microbial populations in the ISS and other closed habitats. Since investigators can only collect samples infrequently from the ISS itself due to logistical reasons, Earth analogs, such as spacecraft-assembly clean rooms, are used and extensively characterized for the presence of microbes. Microbiologists identify the predominant, problematic, and extremophilic microbial species in these closed habitats and use the ISS as a testbed to study their resistance to extreme extraterrestrial environmental conditions. Investigators monitor the microbes exposed to the real space conditions in order to track their genomic changes in response to the selective pressures present in outer space (external to the ISS) and the spaceflight (in the interior of the ISS). In this review, we discussed the presence of microbes in space research-related closed habitats and the resistance of some microbial species to the extreme environmental conditions of space.

## 1. Microbial monitoring in connection with robotic and human space missions

### 1.1. NASA Robotic Exploration and Microbial Monitoring

Through its planetary protection efforts, the National Aeronautics and Space Administration (NASA) wards against (a) forward contamination, which is the contamination of certain solar system bodies by biological material from Earth, and (b) back contamination, the contamination of Earth by harmful materials returned from space. Forward contamination warns the scientific integrity of life-detection space missions. Back contamination threatens the health and safety of life on Earth (40). To prevent forward contamination, NASA requires that all components of the spacecraft intended to land on certain planets such as Mars be assembled in clean rooms maintained at strictly defined standards of cleanliness. Clean rooms are made as inhospitable as possible to microorganisms through methods ranging from disinfectants to sophisticated air filters and temperature and humidity controls. However, some species persist (21). Recent studies repeatedly isolated certain bacterial taxa in spacecraft clean rooms that were fastidiously maintained to the highest industry standards (8, 21, 30, 31). While these studies allowed various extremophiles present in spacecraft-related closed habitats to be identified, further research is needed to predict the risk of these microbes to life-detection missions (46). Future research should determine the distribution of particularly high-risk microbes on spacecraft surfaces (54).

Bacterial endospores have long been considered potentially capable of surviving the conditions of interplanetary travel to Mars and beyond (7, 19, 43). Therefore, NASA has used the incidence of aerobic, heterotrophic, mesophilic, and endospore-forming bacteria as a measure of cleanliness for spacecraft surfaces since 1967. The total number of cultivable bacterial spores detected on a spacecraft must be below a certain maximum allowable limit. This limit is calculated for each particular mission, based on the mission’s purpose, its target planetary environment, and the assessed factor of risk that organisms introduced to that environment would cause, *i.e.*, “harmful (forward) contamination”.

However, bacteria are not the only organisms of concern. Previous studies identified certain species of archaea hardy to simulated Mars-like conditions from spacecraft-associated environments (18, 31). Moreover, recent studies found certain fungal species capable of withstanding extended exposure to space conditions (38). In light of these findings, spacecraft-related microbial assessments need to be broadened to include the Archaea and Eukarya in addition to bacteria. Expanding their scope to all three domains of life, space microbiologists will achieve a more complete assessment of biological diversity on spacecraft surfaces and the forward-contamination risk from the most space-hardy of species detected.

Along with this broadening of scope, various microbial assessment techniques are needed. Not all microbes of interest are amenable to detection via cultivation-based assays. High-throughput pyrosequencing and phylogenetic microarray techniques have recently and markedly increased the ability of investigators to detect a broad spectrum of diverse microbial lineages in spacecraft-associated environments (22, 28, 47). A molecular-based, cultivation-independent method to systematically catalogue microbial genetic signatures associated with spacecraft surfaces will be an important tool for NASA in the next era of solar system exploration, particularly with regards to potential missions to return samples from Mars and explore Jupiter’s moon Europa and other icy satellites. To develop such a method, a Jet Propulsion Laboratory (JPL) team under the aegis of the NASA Mars Program conducted a six-year Genetic Inventory study, and delivered a comprehensive final report in 2012 (51). The team combined three analytical technologies ( conventional cloning techniques, PhyloChip DNA microarrays, and 454 tag-pyrosequencing) with a systematic methodology to collect, process, and archive nucleic acids to assess the phylogenetic breadth of microorganisms on spacecrafts and the associated surfaces (23).

### 1.2. NASA Human Exploration and Microbial Monitoring

While microbial monitoring is important to protect the integrity of scientific results from robotic space missions, it is essential to protect the health and safety of crew members and their environments during human exploration missions. Traditional, culture-based methods have been extensively used to assess the phylogenetic breadth and total microbial burden associated with various closed systems, including the ISS (4), submarines (33, 49), airliner cabins (53), environmental chambers (24, 42), and office buildings (3). Data obtained from the Apollo lunar module, Skylab (48), Space Shuttle (17, 41), and Mir space station (16) revealed that such culture-based studies are invaluable for the construction and maintenance of space environments compatible with human occupation. The findings of culture-based microbiological studies have factored heavily into the design and continued implementation of the ISS. Information gained from such studies has helped keep this closed environment capable of supporting human habitation for many years (41). For example, HEPA filters were incorporated into the air handling system of the ISS to reduce the levels of airborne bacteria, fungi, and particulates (41), and such measures also consistently control the levels of airborne bacteria below the acceptable limit of 1 × 10^4^ colony-forming unit (CFU) m^−3^ (41). However, because these studies were solely based on cultivation, they could not account for the presence of viable, but currently uncultivable microorganisms, a shortcoming that limits current ISS assessments of the total microbial burden. The implementation of state-of-the-art molecular microbiological techniques should reveal the presence of countless viable, but currently uncultivable microbes. This information will aid in the design and development of appropriate cleaning and sterilization countermeasures for use if undesirable levels of microorganisms are detected. In addition, molecular microbial diversity studies on closed systems, such as the ISS, could provide key metrics for establishing criteria to maintain an environment that promotes the health, safety, and productivity of crew members.

Molecular assessments of the microbial diversity of ISS drinking water (20), ISS-integrated thermal coolant system (2), and closed mock-up habitat supporting ISS research (29) recently revealed the presence and, in some cases, even the predominance of certain microbes reported to be harmful to human health (*i.e.*, species of *Afipia*, *Propionibacterium*) and/or the habitat (*i.e.*, species of *Ralstonia*). Culture-based approaches have failed to detect the presence of these non-cultivable opportunistic pathogens and metal-fouling microbes. While a few studies have performed molecular microbial community analyses on air samples collected from clean rooms (36) and hospital surgical rooms (6), to the best of our knowledge, similar studies have not yet been conducted on ISS-like closed environments. The Genetic Inventory capability developed by the JPL/NASA Mars Program team is an effective and feasible strategy to meet the challenges of assessing and managing the effects of microorganisms aboard the ISS and in other long-duration closed habitats (for a full report, see 23, 51).

### 1.3. International Space Station—Microbial Observatory Project

The NRC Committee for the Decadal Survey on Biological and Physical Sciences in Space recommended in 2011 that NASA “*capitalize on the technological maturity, low cost, and speed of genomic analyses and the rapid generation time of microbes to monitor the evolution of microbial genomic changes in response to the selective pressures present in the spaceflight environment* (34).” The committee further indicated that “*microbial species that are uncommon, or that have significantly increased or decreased in number, can be studied (in a microbial observatory) on ISS*.” Numerous challenges are associated with providing a comprehensive assessment of the microbial inventory of the ISS environment and include the identification and standardization of ideal sample-collection and molecular-detection techniques that are amenable to the microgravity environment.

As recommended in the NRC Decadal Survey, NASA recently funded a project, led by the lead author (KV), to establish an ISS Microbial Observatory (ISS-MO), whereby investigators can generate an extensive microbial census of the space station’s surfaces and atmosphere using advanced molecular microbial community analysis techniques, supported by traditional culture-based methods and modern bioinformatic computational modeling (52). The establishment of the ISS-MO will lead to long-term, multigenerational studies on microbial population dynamics, which will, in turn, provide answers to NASA’s guiding questions for space biology, particularly questions concerning the genetically defined development of organisms and the effects of microgravity and increased radiation exposure experienced in spaceflight on biological processes.

The microbial census estimated by the ISS-MO project will offer a significant insight into spaceflight-induced changes in the populations of beneficial and potentially harmful microbes. It will provide both improved understanding of the changes themselves (*e.g.*, by cataloging population changes and mapping/linking these to environmental niches and genomic changes) as well as an insight into practical countermeasures for mitigating risks to crew members and their environments from microbial life in the ISS.

The ISS-MO team will use existing ISS sample-collection technologies to generate an initial microbial census. Following their return to Earth, samples from various ISS modules will be analyzed using standardized technologies from Mars Program–funded projects (51). The ISS-MO project will compile and deliver a database containing genomic sequences and genetic information for all of the microbes encountered within the ISS habitat. Using this data, NASA can accurately and confidently assess the status of microbes affecting ISS crew health and habitat. In addition to providing microbial profiles, the ISS-MO team will identify which microbial taxa pose particular threats to crew health. ISS-MO findings will enable investigators to resolve and mitigate risks applicable to NASA’s Human Research Program.

#### 1.3.1. Microbial Exposure to Outer Space

From the advent of space travel to today, investigators have probed the survivability of microorganisms in outer space to establish the upper limits of the Earth’s biosphere (10, 13). To test the survivability of organisms in habitats independent of Earth’s geography, several space agencies have developed exobiology research facilities whereby investigators can characterize terrestrial organic matter that has been transported to a low Earth orbit or beyond. For example, NASA built the Microbial Ecology Evaluation Device for the Apollo 16 lunar mission; the German Aerospace Center (Deutsches Zentrum für Luft- und Raumfahrt: DLR) developed an Exposure tray mounted on SpaceLab; the Russian counterparts developed exposure platforms for Salut-7, Mir, and Bion; and the European Space Agency (ESA) built several facilities, including BIOPAN, carried on five Russian satellites, and the Exobiology Radiation Assembly, flown on the European Retrievable Carrier (see review 45).

Mission ground reference (MGR) experiments set up to simulate conditions in outer space and on Mars, have facilitated the determination of the final layout of space-borne experimental facilities and have served to clarify phenomena observed in these facilities (1, 12, 35, 37, 45). However, the complex matrix of all (or a selection of) space parameters in microgravity is only available in orbit or beyond. Therefore, the biological effects of space environments, taken as an integrated whole, can only be studied fully *in situ*, *i.e.*, in “real” space conditions.

#### 1.3.2. Microbial Resistance Exhibited by Environmental Strains

The majority of studies published on microbial resistance to space conditions, particularly radiation, have been based on analyses of laboratory strains (9, 11), microbial mats (38), encapsulated extremophiles (25), or epilithic communities (5). As a consequence, researchers currently only have a limited understanding of how terrestrial life exposed in monolayers to real space conditions may be able to survive and possibly adapt. However, particular strains of species may have differing degrees of resistance to challenging environmental conditions. *B. pumilus* SAFR-032, exposed in monolayers to simulated martian UV radiation, exhibited markedly higher resistance than other strains of the same species exposed in the same manner (39). Furthermore, spores of *B. pumilus* SAFR-032 were found to be six times more resistant to UV irradiation and 50 times more resistant to gamma irradiation than the spores of laboratory strain *B. subtilis* 168 (35). Given this evidence of strain-specific resistance, it was important to investigate the resistance of spacecraft-associated environmental isolates such as *B. pumilus* SAFR-032 *in situ* to relevant outer space conditions (50).

## 2. Microbial exposure to outer space

### 2.1. The ESA EXPOSE Facility

**Fig. 1:**
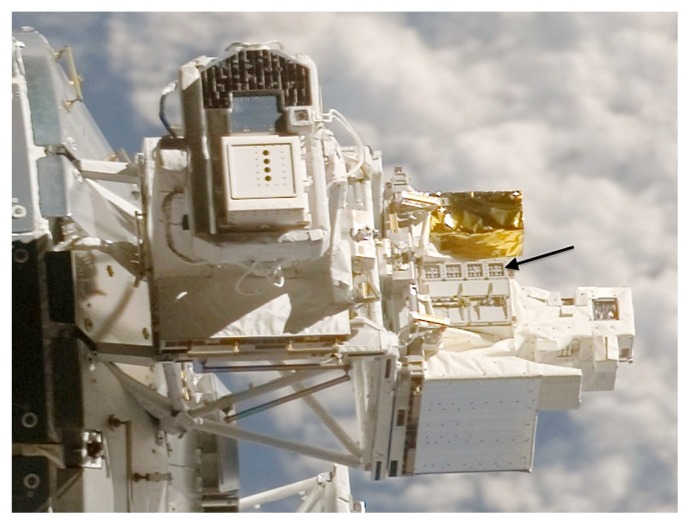
The EXPOSE facility as part of EuTEF attached to the outer platform (balcony) of the Columbus module of the ISS. The arrow shows the EXPOSE facility.

**Fig. 2:**
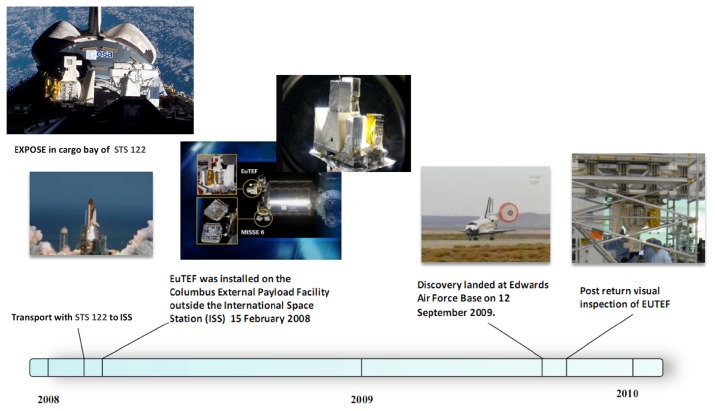
Time line of the EXPOSE-E mission from launch to landing/retrieval.

EXPOSE is an exobiology research facility developed by ESA for medium- to long-term flights on the ISS (45). EXPOSE allows biological and chemical samples to be exposed to outer space while temperature and radiation spectra are recorded and controlled. Mounted in 2008 on an external balcony of the Columbus module on the ISS as part of the European Technology Exposure Facility (EuTEF), the first EXPOSE mission, EXPOSE-E, was used as a testbed for various space conditions (Fig. 1). Between 2008 and 2009, biological and chemical samples were exposed to outer space in the facility for 1.5 years (45). The timeline for the EXPOSE-E mission is shown in Fig. 2.

### 2.2. PROTECT Flight Project (2008–2010)

EXPOSE-E accommodated five astrobiological experiments (44), including the DLR-led PROTECT experiment, which investigated the resistance of various kinds of spores to the open space environment (14). In PROTECT, *B. pumilus* SAFR-032 (50) spores isolated from spacecraft-associated environments and “space-veteran” *B. subtilis* 168 spores (14) were exposed to real-space conditions for a prolonged period of time (~18 months). PROTECT investigators tested the hypothesis that conditions resulting in reduced cellular water content (such as a vacuum and extreme desiccation) may be the main predisposing selective factors for both microbial persistence in interplanetary space and potential proliferation on the martian surface. An MGR experiment simulating flight parameters was conducted in parallel at the Planetary and Space Simulation Facility at DLR.

After 18 months of exposure at the EuTEF EXPOSE-E facility under dark space conditions, ~10 to 40% of *B. pumilus* SAFR-032 spores survived; however, when these spores were kept for the same period under dark and simulated- Mars atmospheric conditions, ~85 to 100% survived. In contrast, when Space UV (>110 nm) was penetrated to SAFR-032 spores for the same time period using EXPOSE, a ~7-log reduction in viability was observed (50). Furthermore, the simulated space conditions on earth were less lethal to spores than real space conditions (Table 1). In the PROTECT experiments, spores of *B. subtilis* 168 survived exposure to outer space. Their survival was mainly attributed to the multilayer manner in which the spores (3.6×10^8^) were exposed, which may have allowed these spores to be shadowed and protected by the top layers (14). In contrast, *B. pumilus* SAFR-032 spores were exposed in a monolayer fashion (1.1×10^7^), and an approximate 7-log reduction in viability was observed. However, a few surviving space-exposed spores exhibited even greater resistance to UV than their MGR counterparts (50).

The complex radiation field experienced in outer space cannot be simulated by any ground facility. The synergistic effects of microgravity and radiation (13) may play an important role in real space conditions and must also be taken into account. The sustainability challenges presented by space conditions for microorganisms also include intense galactic and solar radiation, extreme variations in temperature, microgravity, and a high vacuum. The effects of these environmental extremes in combination with complex spacecraft surfaces with ample shielding opportunities for microorganisms will be the decisive parameters in spore inactivation (32). Hence, *in situ* exposure to outer space is necessary to provide empirical data on microbial survival mechanisms in extraterrestrial environments. However, not all parameters of space can be simulated in the laboratory. This was demonstrated by the survival results shown in Table 1, in which the survival of space flight samples was significantly lower than that of samples treated similarly in the MGR.

Spores that survived real-space conditions in the PROTECT experiment exhibited enhanced UV resistance (~4,000 Jm^2^; UVC) over that of the MGR spores (~2,000 Jm^2^; UVC). Space-surviving spores also exhibited genetic and proteomic changes (50). The second-most-common upregulated protein in the real-space-UV-exposed spores was superoxide dismutase, which has been shown to catalyze the dismutation of superoxide radicals (26, 27). The PROTECT results are important for assessing the limits and mechanisms of microbial survival in extraterrestrial environments, estimating the risks of forward contamination from microbial species, and ensuring the accuracy of *in situ* life-detection investigations in space. However, the following key questions require more in-depth examinations: (i) What is the nature and importance of spore DNA damage in SAFR-032 caused by exposure to a vacuum, solar radiation, simulated Martian surface radiation, and cosmic radiation? (ii) At what rates does this damage accumulate, and how do these accumulation rates correspond to spore inactivation rates? (iii) What specific repair systems are activated during subsequent spore germination? The SAFR-032 spores that were exposed to real-space conditions and the corresponding MGR specimens have been archived at JPL. Future experiments on space-exposed archived samples with the use of emerging molecular techniques will help to further elucidate how and why spacecraft-associated microbes may survive as hitchhikers on space vehicles, thereby potentially compromising the science of life-detection and sample-return missions.

### 2.3. The BOSS Flight Project (2014–)

In the DLR-led Biofilm Organisms Surfing Space (BOSS) project, to be launched in 2014 as part of the EXPOSE-R2 mission on the ISS, investigators will test the hypothesis that biofilm-embedded forms of life survive longer than planktonic forms under the harsh environmental conditions in outer space and on Mars. Particles associated with biofilms may provide additional protection against those conditions (15); therefore, the international BOSS team will expose a range of microorganisms embedded and aggregated in extracellular polymeric substance matrices. The ESA EXPOSE facility, to be attached to the Zvezda module of the ISS, will be used to compare the survivability of different organisms in and out of a biofilm. The molecular and biochemical characterization of microorganisms exposed to real-space conditions will reveal the features of biomolecules and biopolymers that define the physical-chemical limits of life in extreme conditions. The objectives of the BOSS investigation, coupled with *in situ* flight experiments investigating the biology of microorganisms associated with spacecraft surfaces, are to (a) assess the survival rate of microorganisms under space conditions and (b) characterize the biomolecular composition of surviving microorganisms exposed to space conditions compared to controls.

## 3. Conclusion

The results of the ISS-MO project will enable the feasibility of using modern molecular techniques to elucidate microbial changes in closed space habitats such as the ISS. Since an exhaustive catalogue of microbial diversity in the ISS is not yet available, investigators should also devote continued attention to the isolation and characterization of problematic microbial communities. Once ISS microbial communities are isolated, archived, and made available to researchers, appropriate countermeasures to eradicate the problematic microbes can be developed to the benefit of future human spaceflight programs. At present, the ISS provides an invaluable testbed for measuring microbial resilience. Recent experiments in EXPOSE on the ISS have shown that some microbial populations can withstand the extreme environmental conditions of space for markedly long periods. Further spaceflight studies exposing various kinds of extremophiles to outer space will help to reveal the metabolic plasticity that allows these microorganisms to persist, survive, and potentially proliferate in interplanetary space or on other planets. In parallel, a comprehensive state-of-art molecular analysis of microbes in a closed environment on the ground, cataloguing the microbial inventory associated with spacecraft components combined with metabolic and physiological analyses of space-exposed microorganisms, will facilitate in developing suitable microbial burden requirements. Such research will further enable the success of future human space missions.

## Figures and Tables

**Table 1:** PROTECT experiment on the EXPOSE-E mission: Survival of the spores of *B. subtilis* 168 after exposure for 1.5 years to outer space conditions and simulated Mars conditions (Space flight experiment) and after 1.5 years in the Mission Ground Reference (MGR) running in parallel to the flight experiment in the Planetary and Space Simulation facility at the DLR (Reproduced from Horneck *et al.*, Astrobiology, 12, 445–456. 2012).

Conditions	Number of colonies (N)					Survival (N/N_0_)			
	
	Lab control (N_0_; before)a)	Lab control (after 18 months)	Sun exposed 100% Tb)	Sun exposed 0.1% Tb)	Dark space exposedc)	Lab control (after 18 months)	Sun exposed 100% Tb)	Sun exposed 0.1% Tb)	Dark space exposedc)
*Outer space (Space flight)*									
	(3.6±0.5) ×10^8^	(3.9±0.5) ×10^8^	(3.2±2.0) ×10^5^	(3.8±1.7) ×10^6^	(2.0±0.2) ×10^8^	(1.1±0.1)	(8.8±1.4) ×10^−4^	(1.1±0.1) ×10^−2^	(5.5±1.0) ×10^−1^
*Simulated Mars climate (Space flight)*									
	(3.6±0.5) ×10^8^	(3.9±0.5) ×10^8^	(1.8±0.7) ×10^7^	(7.3±0.9) ×10^7^	(2.7±0.4) ×10^8^	(1.1±0.1)	(4.9±0.9) ×10^−2^	(2.0±0.3) ×10^−1^	(7.4±1.3) ×10^−1^
*Simulated space environment (Mission ground reference control)*									
	(3.6±0.5) ×10^8^	(3.9±0.5) ×10^8^	(1.0±0.2) ×10^6^	(1.6±0.3) ×10^7^	(1.8±0.4) ×10^8^	(1.1±0.1)	(2.8±0.6) ×10^−3^	(4.5±0.6) ×10^−2^	(4.8±1.0) ×10^−1^
*Simulated Mars climate (Mission ground reference control)*									
	(3.6±0.5) ×10^8^	(3.9±0.5) ×10^8^	(3.2±0.4) ×10^7^	(6.3±0.8) ×10^7^	(2.4±0.2) ×10^8^	(1.1±0.1)	(9.0±1.2) ×10^−2^	(1.7±0.4) ×10^−1^	(6.7±1.2) ×10^−1^

a) *n* = 5, taken as the untreated control N_0_

b) *n* = 2

c) *n* = 21

T = transmission of sunlight through the optical filter system
